# Recent Updates on Predicting Conversion in Youth at Clinical High Risk for Psychosis

**DOI:** 10.1007/s11920-023-01456-2

**Published:** 2023-09-27

**Authors:** Noe Caballero, Siddharth Machiraju, Anthony Diomino, Leda Kennedy, Armita Kadivar, Kristin S. Cadenhead

**Affiliations:** Department of Psychiatry, University of California San Diego, 9500 Gilman Dr., La Jolla, CA 92093-0810 USA

**Keywords:** Psychosis, Clinical high risk, Prodome, Conversion, Biomarkers, Treatment

## Abstract

**Purpose of Review:**

This review highlights recent advances in the prediction and treatment of psychotic conversion. Over the past 25 years, research into the prodromal phase of psychotic illness has expanded with the promise of early identification of individuals at clinical high risk (CHR) for psychosis who are likely to convert to psychosis.

**Recent Findings:**

Meta-analyses highlight conversion rates between 20 and 30% within 2–3 years using existing clinical criteria while research into more specific risk factors, biomarkers, and refinement of psychosis risk calculators has exploded, improving our ability to predict psychotic conversion with greater accuracy. Recent studies highlight risk factors and biomarkers likely to contribute to earlier identification and provide insight into neurodevelopmental abnormalities, CHR subtypes, and interventions that can target specific risk profiles linked to neural mechanisms.

**Summary:**

Ongoing initiatives that assess longer-term (> 5–10 years) outcome of CHR participants can provide valuable information about predictors of later conversion and diagnostic outcomes while large-scale international biomarker studies provide hope for precision intervention that will alter the course of early psychosis globally.

## Introduction

Foundational research from the past two decades has elucidated the presence of the clinical high risk (CHR) state, or the period prior to the onset of psychosis [1, 2]. This prodromal phase of illness has been referred to as CHR, attenuated psychosis syndrome (APS), and ultra high risk (UHR) and has been studied internationally as a critical time window for early identification and intervention [2–4]. For the purposes of this review, we will refer to this period as the CHR phase and will refer to individuals as CHR to denote this risk for psychosis. Adapted from findings in schizophrenia cohorts, attenuated positive symptoms such as unusual thought content, suspiciousness, and perceptual abnormalities are now understood to exist on a clinical spectrum of severity, and are used as primary metrics to determine if an individual has crossed the “threshold” from CHR to a full-blown psychotic disorder [3, 5, 6]. In the literature, this is widely referred to as “conversion” or “transition” to psychosis, denoted as CHR-C (converted) versus CHR-NC (non-converted) in this review.

Meta-analyses provide estimates of conversion rates between 20 and 30% within 2–3 years among those that meet criteria for CHR [4]. A recent meta-analysis by Salazar de Pablo et al. [7] revealed similar conversion estimates of 25% in a span of 2–3 years, additionally suggesting that risk for conversion to psychosis increases with time. While findings in conversion rates have been comparable over the past several decades, they remain heterogenous, with many studies using different methodologies, definitions, and controlling for different confounders [7–9].

Identifying biomarkers linked to psychotic conversion has become a critical directive in the early psychosis field to not only predict risk of conversion with greater accuracy but to better understand the mechanism of conversion and to identify critical treatment targets linked to neurobiology. Importantly, multi-site large-scale studies have identified epidemiological, neuroimaging, electrophysiological, neurocognitive, inflammatory, genetic, and neurohormonal biomarkers that are associated with increased risk for conversion to psychosis [10].

Despite landmark findings regarding conversion rates and predictors of conversion in prodromal psychosis from the early part of the twenty-first century, there have been few works which have evaluated and summarized these findings in recent years [4, 7, 11]. The aim of this review is to synthesize contemporary research findings regarding prediction of conversion to psychosis among CHR cohorts from 2019 to the present day. This review will highlight areas where critical questions in the pursuit of predicting conversion remain and will provide insight into future research avenues that may improve the fields’ ability to predict the onset of psychosis in CHR youth, to understand the neurobiological mechanisms, and to identify targeted treatments.

## Epidemiological Risk Factors for Psychotic Conversion

A variety of clinical and environmental predictors of conversion to psychosis have been identified within CHR cohorts [12–14]. When combined, these various risk factors have contributed to predictive models and psychosis risk calculators with higher prognostic accuracy for psychosis in CHR than any one alone [15] (see “Prediction Models” below). Earlier and more precise identification of psychosis risk could lead to better-targeted preventive efforts in this population [16]. Past reviews of the literature have identified factors such as poorer social functioning, severity of subthreshold positive symptoms, cannabis use, migrant status, and genetic risk for schizophrenia as consistent predictors of later psychotic conversion [5, 17].

Since 2019, several reports have investigated epidemiological factors linked to psychosis (Table 1). A recent epidemiological study by Bolhuis et al. [18••] assessed individuals born in Finland in 1987 and found that of those who presented to the hospital for self-harm, 12.8% went on to receive a diagnosis of psychosis and 9.4% a diagnosis of bipolar disorder by 28 years of age. The investigators also found that younger age of first self-harm was associated with higher risk of conversion; 29.1% of those who presented with self-harm before the age of 18 developed a psychotic or bipolar disorder [18••].

**Table 1:** Epidemiology updates

**Key publications**	**Sample**	**Key findings**
Bolhuis et al. (2021) [18••]	General population born in Finland in 1987 (*N* = 59,476)	Hospital presentation for self-harm associated with later psychotic or bipolar disorder, of those who presented prior at age 18, 29.1% went on to develop a psychotic or bipolar disorder by age 28
Tronick et al. (2023) [20]	NAPLS3: CHR (*N* = 684), CHR-C (*N* = 68), CHR-NC (*N* = 380)	CHR-C scored lower on the protective factors index, specifically on prosocial involvement and resilient personality traits
Barbato et al. (2022) [19]	NAPLS3: CHR (*N* = 710), CHR-C (*N* = 49), CHR-NC (*N* = 197)	Rates of conversion did not differ across migrant status groups
Salazar de Pablo et al. (2021) [7]	Meta-analysis 130 studies: CHR (*N* = 9222)	Age is not a moderator of transition risk

*NAPLS* North American Prodrome Longitudinal Studies, *CHR* clinical high risk, *CHR-C* clinical high risk converted, *CHR-NC* CHR non-converted

Recent reports have also investigated epidemiologic risk factors within CHR cohorts. Barbato et al. [19] assessed whether migrant status is a predictor of transition to psychosis within the North American Prodrome Longitudinal Study phase 3 (NAPLS3) cohort. No significant difference was found between the migrant status defined groups (native-born, first-generation, or second-generation) in symptoms or functioning at any time point and transition rates did not differ across groups [19]. Tronick et al. [20], also from the NAPLS3 consortium, found that CHR-C scored lower on a protective factors index—including prosocial involvement and resilient personality traits—compared to CHR-NC, while other risk factors also associated with violence risk were not predictive of conversion. Furthermore, while prior studies have found mixed results when assessing age as a risk factor for development of psychosis [21, 22], a recent meta-analysis by Salazar de Pablo et al. [7] found that age did not moderate transition risk.

Overall, studies of epidemiological risk factors for psychotic conversion have identified emergency room visits for self-harm as a risk factor for psychosis in a general population while recent findings on migrant status and age as risk factors among CHR cohorts are less conclusive. Conversion risk has also been linked to fewer protective factors, suggesting that bolstering resilience could enhance preventative efforts. These data suggest that early evidence of self-harm could also be an important risk factor not only in the general population but perhaps in CHR youth and focused intervention efforts in this population may mitigate future risk of serious mental illness [18••, 20].

## Biomarkers Linked to Psychotic Conversion

### Neuroimaging

Recent neuroimaging literature findings support the notion that detectable patterns in brain morphometry and functional neuroanatomy are associated with conversion to psychosis in CHR youth [23••]. In one of the first CHR neuroimaging studies, Pantelis et al. [24] demonstrated that decreased gray matter volume was associated with later psychotic conversion in a cross-sectional design while repeat scans revealed continued reduction in gray matter in CHR-C vs CHR-NC. These early cross-sectional and longitudinal findings were replicated in several subsequent studies [25–27], further highlighting the importance of assessing neuroimaging biomarkers cross-sectionally as well as change over time as a risk factor for psychosis.

Recent studies (Table 2) including those from the Shanghai at Risk for Psychosis (SHARP) [28] and NAPLS3 [23••] cohorts have reported that decreased cortical thickness and accelerated cortical thinning are associated with conversion. Del Re et al. [28] found that decreased relative cortical thickness in the superior temporal sulcus, Heschl’s gyrus, and pars triangularis differentiated the CHR-C from the CHR-NC after 1 year follow-up while Collins et al. [23••] found accelerated thinning across several cortical regions in the prefrontal, temporal, and parietal regions in CHR-C vs CHR-NC.

**Table 2:** Neuroimaging updates

**Key publications**	**Sample**	**Key findings**
**Morphometry studies**		
Del Re et al. (2021) [28]	SHARP: CHR (*N* = 152), CHR-C (*N* = 22), CHR-NC (*N* = 130)	CHR-C vs CHR-NC reduced cortical thickness in the superior temporal sulcus, Heschl’s gyrus, and pars triangularis
Collins et al. (2022) [23••]	NAPLS3: CHR (*N* = 382), CHR-C (*N* = 42), CHR-NC (*N* = 338)	CHR-C vs CHR-NC greater cortical thinning over time in the prefrontal, temporal, and parietal cortical regions
**Diffusion tensor imaging**		
Kristensen et al. (2021) [29]	Denmark: CHR (*N* = 110), CHR-C (*N* = 10), CHR-NC (*N* = 100)	CHR-C vs CHR-NC reduced global FA
Nägele et al. (2021) [30]	Germany: CHR (*N* = 30), CHR-C (*N* = 8), CHR-NC (*N* = 22)	CHR-C vs CHR-NC reduced FA of cellular tissue
León-Ortiz et al. (2022) [31]	Mexico: CHR (*N* = 33), CHR-C (*N* = 7), CHR-NC (*N* = 26)	CHR-C vs CHR-NC differences in FA values in posterior thalamic radiation
**Resting state ofMRI**		
Collin et al. (2020) [34]	SHARP: CHR (*N* = 158), CHR-C (*N* = 23), CHR-NC (*N* = 135)	CHR-C vs CHR-NC abnormal baseline modular connectome organization
Chen et al. (2021) [35••]	NAPLS2: CHR (*N* = 263), CHR-C (*N* = 25), CHR-NC (*N* = 238)	CHR-C vs CHR-NC increased activity in frontoparietal network, inferior temporal gyrus, cerebellum, negative mediators included DMN, thalamus, visual cortex, cerebellar lobe 8
Cao et al. (2019) [36]	NAPLS2: CHR (*N* = 155), CHR-C (*N* = 18), CHR-NC (*N* = 137)	CHR-C vs CHR-NC reduction in global efficiency and an increase in network diversity, primarily driven by DMN
**Proton magnetic resonance spectroscopy studies (**^**1**^**H-MRS)**		
Leon-Ortiz et al. (2022) [31]	Mexico: CHR (*N* = 33), CHR-C (*N* = 7), CHR-NC (*N* = 26)	CHR-C vs CHR-NC no differences in Glu/Glx striatum
Provenzano et al. (2020) [42]	CHR (*N* = 75), CHR-C (*N* = 25), CHR-NC (*N* = 50)	CHR-NC vs CHR-C no differences in hippocampal glu
Bossong et al. (2019) [43]	CHR (*N* = 86), CHR-C (*N* = 12), CHR-NC (*N* = 74)	CHR-C vs CHR-NC greater hippocampal glu, mI, and cr

*SHARP* Shanghai at Risk for Psychosis, *NAPLS* North American Prodrome Longitudinal Studies, *CHR* clinical high risk, *CHR-C* clinical high risk converted, *CHR-NC* CHR non-converted, *FA* fractional anisotropy, *AUC* area under the curve, *DMN* default mode network, *Glu* Glutamate, *Glx* Glutamine + Glu, *mI* myo-inositol, *cr* creatine

White matter alterations measured with diffusion tensor imaging (DTI) and fractional anisotropy (FA) have also recently been explored as potential predictors of psychotic conversion [29–31]. Kristensen et al. [29] demonstrated that a prediction model incorporating FA at baseline assessment predicted conversion to psychosis in a CHR sample from Denmark (see “Prediction Models” below). In alignment with those findings, Nägele et al. [30] observed significantly lower FA in commissural and association tracts in CHR-C vs CHR-NC in a sample from Germany, while León-Ortiz et al. [31] found that lower FA in the posterior thalamic radiation differentiated between CHR-C and CHR-NC in Mexico. These studies suggest that white matter alterations among CHR may be a valid neuroimaging marker for future study in predictive models of psychosis.

Previous studies using resting-state functional connectivity MRI (rs fMRI) have identified thalamocortical and thalamocerebellar dysconnectivity and hyperconnectivity with sensorimotor cortical areas respectively as potential biomarkers of psychosis risk in CHR-C participants [32, 33]. More recently, Collin et al. [34] found that abnormal modular connectome organization at baseline predicted conversion to psychosis as part of the SHARP study. Chen et al. [35••] designed a high-dimensional brain-wide functional mediation framework and used rs fMRI data from the NAPLS2 sample to identify neural markers potentially linked to conversion including increased activity in the frontoparietal network and inferior temporal gyrus and cerebellum as well as negative mediators that were part of the default mode network (DMN), thalamus, visual cortex, and cerebellar lobe 8. Cao et al. [36] investigated longitudinal changes in rs fMRI network from a subsample in NAPLS2 and found that CHR-C showed a reduction in global efficiency and an increase in network diversity relative to CHR-NC and this was primarily driven by the DMN.

Proton magnetic resonance spectroscopy studies (^1^H-MRS) have identified neurometabolic changes in various brain regions that may be unique to the onset of psychosis and provide insight into the neuropathological changes early in the course of illness [37–41]. In one of the first ^1^H-MRS studies addressing conversion in CHR, de la Fuente-Sandoval et al. [37] reported higher glutamate (glu) levels in the striatum in CHR-C compared to CHR-NC. In a recent follow-up report from the same group, Leon-Ortiz et al. [31] did not replicate their previous ^1^H-MRS glu results in a Mexican sample but they observed significant correlations between striatal glu and FA results. Provenzano et al. [42] found that CHR participants had high glu/glx (glu + glutamine) in the hippocampus compared to controls but did not find any association with conversion to psychosis. A recent publication from Bossong et al. [43] in the UK reported that higher levels of hippocampal glutamate predicted conversion along with higher myo-inositol and creatine.

Altogether, neuroimaging has identified several promising imaging biomarkers that may be helpful in both predicting conversion to psychosis as well as conceptualizing structural, functional, and metabolic changes in the brain that precede conversion. One ongoing challenge in neuroimaging and conversion literature is using data-driven approaches to improving existing prediction algorithms and risk calculators (see “Prediction Models” section).

### Electrophysiology

A body of literature supports the notion of impaired sensory and cognitive processing prior to and upon conversion to psychosis. Event-related potentials (ERPs) and sensorimotor gating, measurable by electroencephalogram (EEG) or electromyography (EMG) as stereotyped responses to stimuli, have consistently garnered interest as potential neurobiological biomarkers of clinical outcomes in CHR including conversion risk, owing to their robust findings in psychosis [44]. Prior to 2019, the CHR research community honed in on several measures, including mismatch negativity (MMN), oddball, P50 sensory gating, neural synchrony, and prepulse inhibition (PPI) paradigms as potential predictors of psychotic conversion [44–48]. Several early studies highlighted that a reduced P300 amplitude in oddball paradigms was predictive of imminent psychosis [49, 50]. These findings, taken together, have propelled rigorous investigation of each paradigm as objective, measurable biomarkers of conversion.

Since 2019, several important ERP papers have been published, further contributing to the conversion prediction literature in electrophysiology (Table 3). In an auditory oddball paradigm, Hamilton et al. [51••] reported that, among CHR individuals enrolled in the NAPLS2 study, a greater reduction in P300 amplitude—in particular, a deficit in target P3b amplitude—was associated with progression to psychosis and implicated a shorter time to conversion [51••], while Tang et al. [52] reported that reduced novel P3a amplitude was predictive of conversion in a Chinese cohort. Foss-Feig et al. [53] expanded on this work within a NAPLS2 sub-cohort of CHR with comorbid autism spectrum disorder (ASD), given higher rates of psychosis in ASD compared to the general population. Of note, the investigators not only reported that P300 amplitude differentially predicted conversion to psychosis among CHR, but also that comorbid ASD moderated this relationship [53]. Though prior literature supports the association between a smaller P300 amplitude and conversion to psychosis, their findings suggested that a greater P300 amplitude was associated with conversion among CHR with a history of ASD [53]. Within the realm of sensory registration, a recent study by Duncan et al. [54] from the NAPLS2 consortium reported that a reduction in N100 amplitude measured in the auditory oddball task was predictive of conversion to psychosis in CHR. The investigators found that a smaller N100 amplitude in response to both standard and novel stimuli was predictive of conversion to psychosis [54]. Furthermore, a smaller N100 amplitude was associated with shorter time to conversion for both standard and novel stimuli [54].

**Table 3:** Electrophysiology updates

**Key publications**	**Sample**	**Key findings**
**P300 oddball paradigm**		
Hamilton et al. (2019) [51••]	NAPLS2: CHR (*N* = 552), CHR-C (*N* = 73), CHR-NC (*N* = 225)	CHR-C vs CHR-NC smaller auditory target P3b amplitude and a shorter time to conversion
Tang et al. (2020) et al. [52]	SHARP: CHR (*N* = 104), CHR-C (*N* = 19), CHR-NC (*N* = 75)	CHR-C vs CHR-NC smaller auditory novel P3a
Foss-Feig et al. (2021) [53]	NAPLS2: CHR (*N* = 304, 14 ASD + , 290 ASD-), CHR-C (*N* = 75, 4 ASD + , 71 ASD-)	CHR-C vs CHR-NC smaller visual novel P3a amplitude and auditory target P3b amplitude but comorbid ASD moderated this relationship and greater P300 amplitudes were associated with conversion among CHR + ASD individuals
Duncan et al. (2022) [54]	NAPLS2: CHR (*N* = 552), CHR-C (*N* = 73), CHR-NC (*N* = 225)	CHR-C vs CHR-NC had reduced N100 amplitude to both standard and novel stimuli that was associated with earlier time to conversion
**Mismatch negativity**		
Fryer et al. (2020) [56]	NAPLS2: CHR (*N* = 579), CHR-C (*N* = 77), CHR-NC (*N* = 238)	CHR-C vs CHR-NC-Remitted had deficits in response to late-appearing standards. In CHR-C, greater reduction in RP was predictive of shorter time to conversion among those not receiving pharmacotherapy
Hamilton et al. (2022) [55••]	NAPLS2: CHR (*N* = 580), CHR-C (*N* = 77), CHR-NC (*N* = 238)	CHR-C vs CHR-NC had greater deficits in MMN amplitude in double deviant paradigm that was also associated with shorter time to conversion
**Startle modulation**		
Cadenhead et al. (2020) [64]	CHR (*N* = 543), CHR-C (*N* = 58), CHR-NC (*N* = 255)	CHR-C vs CHR-NC had slower startle response latency but did not differ in PPI. In CHR-C, PPI was positively correlated with age while this was not present in HC

*SHARP* Shanghai at Risk for Psychosis, *NAPLS* North American Prodrome Longitudinal Studies, *CHR* clinical high risk, *CHR-C* clinical high risk converted, *CHR-NC* CHR non-converted, *RP* repetitive positivity, *HC* healthy comparison

Newer developments in repetition positivity (RP)—another component of predictive coding—and mismatch negativity (MMN) have also surfaced in recent years. Hamilton et al. [55••] reported that, among CHR not receiving antipsychotics at baseline, an attenuated MMN amplitude in a double deviant paradigm was associated with both conversion to psychosis and decreased time to conversion. Fryer et al. [56] determined that CHR-C had greater deficits in response to late-appearing standards compared to CHR-NC whose symptoms had remitted in the NAPLS2 cohort. The group also observed that a greater reduction in RP was predictive of shorter time to conversion among those not receiving pharmacotherapy [56].

PPI of the startle response is an index of sensorimotor gating that has been shown to be deficient in individuals in the psychosis spectrum [57–59], CHR [60, 61], and translational models of psychosis [62, 63]. Prior to 2019, only one study [46] assessed PPI in CHR participants who later converted to psychosis. Cadenhead [46] found that a small sample of CHR-C had greater PPI than CHR-NC. Since 2019, Cadenhead et al. [64] have published on a larger cohort from the NAPLS2 sample and did not find any PPI differences between CHR-C and CHR-NC but, within the CHR-C sample, age was significantly correlated with PPI (greater with advancing age and not typical of normally developing adolescents), replicating a previous age finding [46], that provided evidence of neurodevelopmental differences in the sample who later converted to psychosis. In addition, the startle response latency, a measure of neural processing speed, was greater in CHR-C compared to CHR-NC, with greater predictive power than clinical symptoms in predicting future psychosis in female CHR. It is therefore possible that slow neural processing represents a potential biomarker of psychosis risk in female CHR. Both the PPI developmental findings and startle latency can be studied in translational models, perhaps providing further insight into brain changes that predict future psychosis.

In summary, research in electrophysiological biomarkers has continued to flourish in the last few years, with considerable traction gained in the study of P300, MMN, N100, and startle latency as predictors of conversion to psychosis. While no single neurophysiologic biomarker is claimed to be a hallmark prognostic marker, multiple measures of information processing may collectively provide insight in the prediction of conversion among CHR.

### Neurocognition

Neurocognitive deficits are prominent across the psychosis spectrum [65–68], are apparent in childhood in those individuals who go on to develop schizophrenia, and tend to exacerbate before the onset of psychotic symptoms [69]. Early reports in CHR samples [67, 70–72] demonstrated neurocognitive deficits across multiple domains that are greatest in CHR-C. Early longitudinal studies also found a decline in neurocognitive domains such as verbal memory over time, in CHR-C [73, 74]. Larger collaborative studies [75, 76] and meta-analyses [77–79] later confirmed the association of neurocognitive deficits with conversion to psychosis and incorporated specific neurocognitive tests (e.g., processing speed and verbal learning and memory) into psychosis risk calculators [21] that, along with clinical and demographic data, predict psychotic conversion with greater accuracy.

Since 2019, several new meta-analyses have been published that confirm not only baseline differences [80, 81] between CHR-C versus CHR-NC but also longitudinal changes [82] and variability [83] of cognitive performance (Table 4). Millman et al. [80] reported that the domains of global cognition, processing speed, and working memory differentiated CHR-C vs CHR-NC, while Catalan et al. [81] identified verbal learning and memory as most associated with transition to psychosis. Hedges et al. [82] examined longitudinal changes and found that CHR participants, like controls, improved over time but CHR-C showed less improvement or a decline in performance on processing speed tasks compared to CHR-NC. Catalan et al. [83] evaluated within-group variability across neurocognitive domains in CHR participants and found that CHR-C showed greater variability in executive functioning compared to CHR-NC.

**Table 4:** Neurocognitive updates

**Key publications**	**Sample**	**Key findings**
**Meta-analyses**		
Millman et al. (2022) [80]	21 studies: CHR (*N* = 482–948), CHR-C (*N* = 42–107), CHR-NC (*N* = 235–557)	CHR-C vs CHR-NC differences in global cognition, processing speed and working memory
Catalan et al. (2021) [81]	78 studies: CHR (*N* = 119–1973), CHR-C (*N* = 37–278), CHR-NC (*N* = 104–1075)	CHR-C vs CHR-NC differences in verbal learning and memory
Hedges et al. (2022) [82]	13 studies: CHR (*N* = 94–431), CHR-C (*N* = 34–86), CHR-NC (*N* = 83–347)	CHR-C vs CHR-NC showed less improvement or a decline in performance in processing speed over time
Catalan et al. (2022) [83]	78 studies: CHR (*N* = 5162)	CHR-C vs CHR-NC showed a greater variability ratio in executive functioning
**Individual and consortia studies**		
Cui et al. (2020) [84]	SHARP: CHR (*N* = 196), CHR-C (*N* = 41), CHR-NC (*N* = 155)	CHR-C vs CHR-NC performed worse in processing speed and visual learning
Luo et al. (2021) [86]	Chinese college students: CHR (*N* = 115), CHR-C (*N* = 29), CHR-NC (*N* = 78)	CHR-C exhibited poorer performance in visual learning, working memory, reasoning, and problem solving compared to non-converters
Zhang et al. (2022) [85]	SHARP: CHR-C (*N* = 43 adolescents, *N* = 34 adults), CHR-NC (*N* = 146 adolescents, *N* = 102 adults)	Adolescent CHR-C vs CHR-NC worse in speed of processing, working memory, verbal learning, visual learning and reasoning and problem solving, adult CHR-C vs CHR-NC worse in visuospatial memory test
**Novel analytic techniques of neurocognitive and psychosis risk data**		
Velthorst et al. (2019) [87]	NAPLS1: CHR (*N* = 166), CHR-C (*N* = 54), CHR-NC (*N* = 112)	Hierarchical clustering derived neurocognitive subgroups. Subgroup with significant neurocognitive impairment had the greatest deficits in processing speed and memory tasks and greatest risk of psychotic conversion (58%) compared to mildly impaired (24%) or normal/high performance (10.3%) subgroups
Haddad et al. (2022) [88]	Brazil: CHR (*N* = 92), CHR-C (*N* = 15), CHR-NC (*N* = 77)	Latent profile analysis identified 4 classes. Class with low neurocognitive performance and decreased expression of emotion was more likely to convert to psychosis
Zhang et al. (2020) [91]	SHARP: CHR (*N* = 289), CHR-C (*N* = 54), CHR-NC (*N* = 235)	3 Subtypes derived from canonical correlation/hierarchical cluster analyses. Subtype with negative symptoms and neurocognitive deficits had the highest risk for psychosis (39% vs 11.1% and 18.6%)
Kim et al. (2019) [90]	CHR (*N* = 60), CHR-C (*N* = 13)	Factor analyzed psychosis risk factors and neurocognitive factor (verbal memory, attention/working memory, psychomotor speed, executive function and spatial memory) was the most predictive of later conversion

*NAPLS* North American Prodrome Longitudinal Studies, *CHR* clinical high risk, *CHR-C* clinical high risk converted, *CHR-NC* CHR non-converted

Recent international studies have replicated the neurocognitive findings in CHR-C [84–86]. As part of the SHARP study [84], CHR-C showed greater deficits in processing speed and visual learning relative to CHR-NC while Zhang et al. [85] found different patterns in adolescents vs adults. Luo et al. [86], in a sample of 115 college students, similarly reported that CHR-C exhibited poorer performance in visual learning, working memory, reasoning, and problem solving compared to CHR-NC.

Several publications have used novel analytic methods to identify neurocognitive subtypes as a means of parsing the heterogeneity among CHR [87–90]. Velthorst et al. [87], using hierarchical clustering on NAPLS1 data, found that the subgroup with significant neurocognitive impairment had the greatest risk of psychotic conversion (58%) compared to mildly impaired (24%) or normal/high performance (10.3%) subgroups. Similarly, Haddad et al. [88] performed a latent profile analysis and found that the class with low neurocognitive performance and decreased expression of emotion was more likely to convert to psychosis. Zhang et al. [91], using canonical correlation and hierarchical cluster analyses, found that the subtype characterized by negative symptoms and cognitive deficits had the highest risk for psychosis. Kim et al. [90] analyzed multiple psychosis risk factors and found that the neurocognitive factor was the most predictive of later conversion.

Taken together, neurocognitive deficits (both at baseline and longitudinally) are a robust predictor of psychotic conversion in cross-cultural CHR populations. Neurocognitive performance, when combined with symptom and demographic risk factors for psychosis, increases the predictive power of psychosis risk calculators with potential utility in identifying CHR subtypes with varying degrees of risk and individualized treatment needs.

### Fluid Biomarkers

Immune, neuroendocrine, and metabolic dysregulation are likely linked in the pathophysiology of psychotic disorders [92–94]. Importantly, groundbreaking studies have explored how various fluid biomarkers linked to these domains and genetics may influence psychotic illness and whether they may help to elucidate and predict future psychotic illness in CHR participants (Table 5) [95–100].

**Table 5:** Fluid biomarkers updates

**Key publications**	**Sample**	**Key findings**
Ouyang et al. (2022) [102]	China: CHR (*N* = 49), CHR-C (*N* = 14), CHR-NC (N = 35)	CHR-C vs CHR-NC higher concentrations of IL-1β and TNF-β
Zhang et al. (2022) [103]	SHARP: CHR (*N* = 84), CHR-C (*N* = 16), CHR-NC (*N* = 68)	CHR-C vs CHR-NC pattern of Th1/Th2 cytokine imbalance (decreased IL-1β and decreased IL-1β/IL-6 ratio)
Dickens et al. (2021) [107]	EU-GEI: CHR (*N* = 263), CHR-C (*N* = 50), CHR-NC (*N* = 213)	CHR-C vs CHR-NC lower baseline ether phospholipid levels
Li et al. (2022) [99]	SHARP: CHR (*N* = 90), CHR-C (*N* = 23), CHR-NC (*N* = 67)	CHR-C vs CHR-NC elevated 1-Stearoyl-2-arachidonoyl-sn-glyceral
Perkins et al. (2020) [111••]	NAPLS2: CHR (*N* = 764), CHR-C (*N* = 80), CHR-NC (*N* = 248)	CHR-C vs CHR-NC PRS was higher in the European sample

*SHARP* Shanghai at Risk for Psychosis, *NAPLS* North American Prodrome Longitudinal Studies, *EU-GEI* European Network of National Schizophrenia Networks Studying Gene-Environment Interactions, *CHR* clinical high risk, *CHR-C* clinical high risk converted, *CHR-NC* CHR non-converted

Perkins et al. [98], in their study utilizing a plasma biomarker assay, found that 15 largely immunomodulatory and neurohormonal biomarkers helped distinguish CHR-C from CHR-NC in the NAPLS2 sample. While a recent meta-analysis [101] found no significant trends in inflammatory biomarkers levels in CHR-C vs CHR-NC, studies since continue to identify potential immunomodulatory biomarkers. In a recent study by Ouyang et al. [102], CHR-C had higher levels of TNF-β and IL-17 than CHR-NC, again suggesting that immune dysregulation may characterize psychotic conversion. Zhang et al. [103] investigated whether an imbalance of Th1 and Th2 cytokines was linked to conversion risk, finding that lower IL-1β coupled with a decreased IL-1β/IL-6 ratio was associated with an increased risk of conversion among CHR participants from the SHARP study. Linked to immune dysregulation is hypothalamic–pituitary–adrenal axis dysfunction and in a follow-up to the original report by Walker et al. [104], Worthington et al. [105] reported that higher levels of salivary cortisol predicted psychotic conversion in the NAPLS2 cohort and found including cortisol in the NAPLS Psychosis Risk Calculator improved its predictive accuracy (see “Prediction Models” section below).

The increased prevalence of cardiometabolic abnormalities in antipsychotic naive CHR populations has been described [106], and recent studies have looked at metabolic markers for CHR conversion. In a European Network of National Schizophrenia Networks Studying Gene-Environment Interactions (EU-GEI) study population [107], a machine learning model distinguished between CHR-NC and CHR-C based on a baseline serum lipid profile, with ether phospholipids in particular being at lower levels in CHR-C. Li et al. [99] used a metabolomic approach to identify potential biomarkers and found that changes in unsaturated fatty acid synthesis and elevated 1-stearoyl-2-arachidonoyl-sn-glycerol plasma concentration characterized CHR-C.

Similar to metabolomic approaches, proteomic studies facilitate the discovery of potential biomarkers for psychiatric illness [108]. Mongan et al. [109] utilized proteomic data in an EU-GEI CHR cohort to develop models that were able to effectively predict conversion. In this study, proteins involved in the complement system and coagulation cascade were differentially expressed in participants who converted to psychosis, in line with prior evidence of immune dysregulation and inflammation influencing conversion [109].

Given that there is not one genetic locus that has a large influence on the development of psychotic illness, polygenic risk scores (PRS) have been developed utilizing genome-wide association studies to quantify combined genetic susceptibility for an illness [110]. Perkins et al. [111••] utilized a PRS in CHR in the NAPLS2 sample and found that in the European participants, the PRS was higher in CHR-C compared to CHR-NC, whereas for non-Europeans, no such difference was found; adding this study’s PRS to the NAPLS Psychosis Risk Calculator enhanced the prediction of individual risk (see “Prediction Models” section below).

## Prediction Models

Determining individual risk for conversion to psychosis remains an important challenge in psychiatry, as it has major public health implications. In 2016, Cannon et al. [21] published their work on the Psychosis Risk Calculator developed from the NAPLS2 cohort, using clinical, demographic, and neurocognitive variables—increased unusual thought content and suspiciousness, reduced social functioning, diminished processing speed, decreased verbal learning and memory performance, and younger age at baseline—to predict conversion using time-to-event analysis. Their model determined that the 2-year conversion risk among CHR subjects was 16% with a concordance index (C-index) of 0.71, suggesting good discrimination. The NAPLS Psychosis Risk Calculator was the first of its kind and set forth a push for replication, more rigorous variable selection, the addition of biomarkers, and improved model performance [112–115].

Since 2019 (Table 6), several innovative studies have been done that not only validate existing models but aim to improve on the predictive power of psychosis risk calculators using new technologies and analytic techniques. As part of the Harmonization of At Risk Multisite Observational Networks for Youth (HARMONY) collaboration, Koutsouleris et al. [116] tested the generalizability and prognostic value of the NAPLS Psychosis Risk Calculator in the Personalised Prognostic Tools for Early Psychosis Management (PRONIA) cohort and found good prediction after model calibration to account for sample differences. Zhang et al. [117] developed the SHARP Risk Calculator (SHARP-RC) that used a convenient smartphone-based tool along with clinical predictors and found excellent discriminatory accuracy for psychotic conversion that was then replicated in an independent sample. Ciarleglio et al. [14] developed a prediction model that identified visual perceptual abnormalities, dysphoric mood, unusual thought content, disorganized communication, and violent ideation as having the largest effect sizes. Brodey et al. [118] developed and validated the Early Psychosis Screener for Internet (EPSI) that utilized Support Vector Machine (SVM) classifiers. The EPSI tool when combined with the Structured Interview for Psychosis Risk Syndromes (SIPS) increased the combined positive predicted value of the model [118]. A transdiagnostic prediction model previously developed by Fusar-Poli et al. [115] was readapted and applied to a US electronic health record (EHR)–based study of over 2 million subjects, resulting in a C-index of 0.68 and suggestive of transportability to a distinct population abroad [119]. Due to its potential for clinical utility as evidenced by repeated external validation in multiple settings, the EHR tool has been piloted for use in clinical practice within the UK [119].

**Table 6:** Prediction model updates

**Key publications**	**Sample (training set)**	**Key findings**
Brodey et al. (2019) [118]	CHR (*N* = 182), CHR-C/FEP (*N* = 76), CHR-NC (*N* = 106)	The EPSI-SR tool achieved a PPV of 86.6% when combined with clinician-administered SIPS in differentiating psychosis
Ciarleglio et al. (2019) [14]	CHR (*N* = 199), CHR-C (*N* = 64), CHR-NC (*N* = 135)	Visual perceptual abnormalities, dysphoric mood, unusual thought content, disorganized communication, and violent ideation predicted conversion in the model, C-Index = 0.73
Kegeles et al. (2020) [120]	CHR (*N* = 19), CHR-C (*N* = 7), CHR-NC = 12	Striatal glutamate ^1^H MRS and visual perceptual abnormalities performed with an AUC of 0.87 in a multivariate regression model
Zhang et al. (2019) [113]	SHARP: CHR (*N* = 196), CHR-C (*N* = 51) at 24 months	The smartphone-based SHARP-RC achieved high discriminatory accuracy of predicting conversion to psychosis using four clinical predictors, AUC of 0.78
Kristensen et al. (2021) [29]	Denmark: CHR (*N* = 110), CHR-C (*N* = 10)	Global FA in a multivariate prediction model was predictive of conversion after 12 months (sensitivity 0.70, specificity of 0.88, AUC of 0.87)
Worthington et al. (2020) [131]	NAPLS2: CHR (*N* = 417), CHR-C (*N* = 54) at 24 months	Inclusion of salivary cortisol into the original eight-predictor NAPLS Psychosis Risk Calculator improved its predictive accuracy by 7%, C-index 0.78
Mongan et al. (2021) [109]	EU-GEI: CHR (*N* = 133), CHR-C (*N* = 49), CHR-NC (*N* = 84)	Model included proteomic and clinical predictors AUC 0.95
Dickens et al. (2021) [107]	CHR (*N* = 263), CHR-C (*N* = 50), CHR-NC (*N* = 213)	CHR-C vs CHR-NC distinguished based on lipid profile in model with AUC 0.81 (95% confidence interval = 0.69–0.93)
Koutsouleris et al. (2021) [116]	PRONIA: CHR (*N* = 167), ROD (*N* = 167), CHR-C (*N* = 23), CHR-NC (*N* = 144), ROD-C (*N* = 3), ROD-NC (*N* = 164)	Alongside clinician input, model consisting of structural MRI, schizophrenia PRS, clinical and neurocognitive predictors achieved a balanced accuracy of 85.5% in predicting conversion among CHR and ROD
Cadenhead et al. (2020) [64]	NAPLS2: CHR (*N* = 543), CHR-C (*N* = 58), CHR-NC (*N* = 255)	CHR-C vs CHR-NC had slower startle response latency that was more predictive of conversion than clinical symptoms (AUC 0.65 vs 0.55) in female CHR participants
Perkins et al. (2020) [111••]	NAPLS2: CHR (*N* = 764), CHR-C (*N* = 80), CHR-NC (*N* = 248)	Incorporating PRS into NAPLS psychosis risk calculator contributed 15% risk prediction in Europeans and 7% in non-Europeans

*SHARP* Shanghai at Risk for Psychosis, *NAPLS* North American Prodrome Longitudinal Studies, *EU-GEI* European Network of National Schizophrenia Networks Studying Gene-Environment Interactions, *PRONIA* Personalised Prognostic Tools for Early Psychosis Management, *CHR* clinical high risk, *CHR-C* clinical high risk converted, *CHR-NC* CHR non-converted, *ROD* recent onset depression, *ROD-C* ROD converted, *ROD-NC* ROD non-converted, *PPV* positive predictive power, *PRS* polygenic risk score

Many of the biomarkers described previously in this review have also been incorporated into psychosis risk calculators to improve predictive power. Collins et al. [23••] found that percent cortical thickness change in the left hemisphere performed well in a predictive model from the NAPLS3 consortium differentiating CHR-C vs CHR-NC. In a small sample, Kegeles et al. [120] developed a model with striatal glutamate ^1^H MRS and visual perceptual abnormalities in the Columbia Risk Calculator and found a high area under the curve (AUC). The PRONIA study employed a multimodal machine learning model including structural MRI and psychosis polygenic risk scores, in addition to clinical and neurocognitive predictors, to predict conversion among CHR individuals [121]. They demonstrated that clinician-based classification had a higher specificity, whereas their model was highly sensitive; however, combined human–machine classification had a balanced accuracy in predicting conversion [121]. Kristensen et al. [29] incorporated global FA into a multivariate prediction model finding excellent sensitivity, specificity, and AUC. Dickins et al. [107] used a machine learning approach to develop a model using serum lipids and was able to differentiate CHR-C from CHR-NC groups. Cadenhead et al. [64] added startle response latency to the clinical symptoms used in the NAPLS Psychosis Risk Calculator and found that in female CHR startle latency had a higher AUC than the clinical symptoms in predicting psychosis. Furthermore, Worthington et al. [105] included salivary cortisol in the NAPLS Psychosis Risk Calculator and achieved a good C-index. As previously noted, Mongan et al. [109] developed a well-performing model that incorporated proteomic and clinical data of individuals sampled from the EU-GEI and the Avon Longitudinal Study of Parents and Children samples. Perkins et al. [111••] added the PRS to the NAPLS Psychosis Risk Calculator and found that, with the exception of clinical symptoms, the PRS contributed as much or more than other variables in the calculator in predicting conversion and was significantly correlated with the two neurocognitive domains—processing speed and verbal memory—that are part of the calculator.

Over the last few decades, advancements in technology and predictive models have offered new approaches to predicting risk of conversion among CHR individuals. Since the emergence of COVID-19, digital psychiatry, in particular, has rapidly evolved as a field, with pilot studies and interventions adopting new technologies in both mental health care and research [122]. Various data types and modalities, spanning passive smartphone sensing to self-reported data collection via mobile device apps, have been utilized to study behavior and cognition [123], measure symptom burden [124, 125], and predict early stages of relapse [126–129] among individuals with established psychotic disorders. However, digital phenotyping of CHR individuals prior to developing first-episode psychosis (FEP) remains poorly characterized [130].

With a treasure trove of clinical and biomedical data available, discussion of best practices for building diagnostic and prognostic models is vital. Issues with data-driven research include the use of multivariable models that may not be informed by a priori selection of predictors stemming from clinical and epidemiologic expertise, as well as limited statistical power due to small sample sizes, thereby negatively impacting the predictive accuracy of statistical models [131]. Attention must also be directed toward identifying predictors of remission among CHR individuals who do not experience FEP [132], as well as more efficacious interventions for those eventually identified as high-risk for conversion [100]. Lastly, considerable variability in patient samples, clinical presentations, quantitative methods, and sociocultural contexts complicates the implementation of models in psychiatric practice [133]. However, improved predictive capability of models within recent years has encouraged translation of promising models, but to fully understand their utility in clinical care, pursuit of net benefit analyses is recommended.

## Interventions

While the primary focus of intervention research for CHR populations in recent years has been to synthesize knowledge of treatments which effectively address symptoms and functioning, since 2019 several studies have reviewed the literature on interventions in the context of conversion to psychosis and there have been several clinical trials (Table 7) [134–137]. Interventions that have historically been used to treat symptoms in CHR populations include cognitive behavioral therapy (CBT), low-dose antipsychotic medication, other medication interventions for comorbid symptoms, anti-inflammatory interventions, and cognitive remediation [136, 138]. Devoe et al. [137] performed a systematic review and meta-analysis to evaluate interventions focused on conversion to psychosis. They found that there was a reduced risk for conversion favoring CBT at 12 and 18 months but no interventions were significantly more effective at reducing conversions compared with all other interventions in network meta-analyses [137]. As part of a Cochrane Review [136], Kuharic et al. compared transition rates across different interventions and found no discernable treatment effects on conversion, with the exception of a slightly lower conversion rate among CHR individuals taking Omega-3 supplements compared to placebo in a single study [139]. The Omega-3 trial was repeated as part of the NEURAPRO trial by McGorry et al. [140], but this initially promising finding was unable to be replicated. In a comprehensive meta-analysis of interventions for CHR with a primary outcome of transition to psychosis, Mei et al. [135] found the pooled effect of CBT on the prevention of psychosis at 12 months to be significantly greater than that of comparable interventions, further emphasizing the therapeutic efficacy of CBT to reduce symptoms and possibly prevent the onset of psychosis among CHR youth. In a naturalistic study design in CHR participants who were more symptomatic, Zhang et al. [141] examined the effect of antipsychotic medication on reducing risk of psychotic conversion and found no difference in the conversion rate among those taking antipsychotic medication versus those who were not. Despite much effort, there is little consensus on effective interventions to prevent transition to psychosis in CHR samples [138].

**Table 7:** Intervention updates

**Key publications**	**Sample**	**Key findings**
Devoe et al. (2020) [137]	Meta-analysis: 38 studies	CBT associated with reduction in conversion
Mei et al. (2021) [135]	Meta-analysis: 26 studies	CBT was associated with a reduction in incidence at 12 months
Kuharic et al. (2019) [136]	Cochrane Review: 20 studies	No clear differences between treatments in prevention of conversion, small evidence of Omega-3 in preventing transition to psychosis but low statistical power
Zhang et al. (2022) [141]	SHARP: CHR (*N* = 210), CHR-C (*N* = 56), CHR-NC (*N* = 154)	Antipsychotic treatment (*N* = 151) vs no antipsychotic treatment (*N* = 59) had no effect on conversion rate in a naturalistic design

*SHARP* Shanghai at Risk for Psychosis, *CHR* clinical high risk, *CHR-C* clinical high risk converted, *CHR-NC* CHR non-converted, *CBT* cognitive behavioral therapy

Several studies geared toward trialing interventions in CHR or creating space to test new interventions are fast emerging across the globe [134, 142]. This movement is evident in the funding of major recent research initiatives such as the NIMH biomarker–based research consortia Accelerating Medicines Partnership Program-Schizophrenia (AMP-SCZ) that brings together international researchers to develop better prediction models for conversion and will provide a platform to test novel interventions [143, 144]. The Psychosis Risk Outcomes Network (PRONET) study is a branch of the initiative that aims to map biomarkers, clinical and neuropsychological phenotypes of CHR onto clinical outcomes including conversion to test predictive models and trajectories of CHR [143]. Consistent with the Research Domain Criteria (RDoC) approach to studying psychiatric disorders, recent field-wide emphasis has been placed on identifying modifiable biological treatment targets for CHR as well as on developing interventions that reduce risk for conversion across diverse CHR populations [145]. Predictive models described above may assist in identifying those individuals at elevated risk for conversion who may be appropriate for future treatment studies and clinical treatment trials.

## Conclusions

CHR research over the past few decades has provided important insights into (i) risk factors for conversion to full psychotic illness within a 2–3-year period, (ii) the development of psychosis risk calculators [21], (iii) biomarkers linked to psychosis risk [98, 104, 146, 147], and (iv) evidence of dynamic brain changes [36] that are likely present before the onset of illness and continue to evolve into FEP and more chronic forms of psychosis. Despite these advances in our understanding of the CHR state, longer-term outcomes (5 + years), including eventual diagnoses, have been seldom investigated in the CHR population. Long-term follow-up of CHR individuals provides a unique and rare opportunity to investigate the full trajectory of illness from CHR to first episode to chronic illness.

The CHR criteria identify a heterogeneous population with not only sub-syndromal psychotic symptoms but neurocognitive deficits, comorbid mood, anxiety, and trauma-related symptoms, along with significant social and role functioning problems [148]. Meta-analyses show that 20–30% [149] develop psychosis within 2 years and one-third of known psychotic conversions occur after 2 years [150]. The question of how many conversions occur after 5 years has not been extensively studied in a prospective longitudinal follow-up design. Retrospective studies suggest that the prodromal phase of illness can last up to 20 years [151], but it is unclear which early CHR characteristics predict a later vs early psychotic conversion, affective vs non-affective psychosis, or good vs poor functional outcome.

Substantial evidence already exists for multiple biomarker abnormalities in CHR [76, 98, 104, 147, 152–154]. Specifically, CHR youth show deficits in neurocognition [76], regional cortical gray matter [153], and ERP amplitudes [147, 154], as well as higher PRS [152], inflammatory markers [98], and cortisol [104], relative to comparison subjects. Biomarkers also predict who will convert to psychosis [104, 147, 153, 154] at 2 years and add to the predictive power of psychosis risk calculators.

With NIMH initiatives such as AMP-SCZ, it will be possible to bring together the rapidly developing research in biomarkers and prediction algorithms to investigate treatments linked to the identified neurobiological mechanisms and perhaps individualize interventions based on each person’s unique biological signature.
